# Psychological and functional factors associated with quality of life in comprehensive geriatric care: evidence from a multicenter observational cohort study

**DOI:** 10.1186/s12877-026-07728-9

**Published:** 2026-05-28

**Authors:** Sarah Mendorf, Aline Schönenberg, Konstantin G. Heimrich, Tino Prell

**Affiliations:** Department of Geriatrics, Jena University Hospital, Jena, Germany

**Keywords:** Geriatric care, Quality of life, Psychosocial factors, Functional abilities, Multicenter study

## Abstract

**Background:**

Quality of life (QoL) serves as a central patient-reported outcome but is insufficiently integrated into Comprehensive Geriatric Care (CGC). Although CGC includes a standardized Comprehensive Geriatric Assessment (CGA) to evaluate patient’s initial mobility, cognition, depression, and functional impairment, it remains unclear whether it adequately addresses the factors most relevant to QoL.

**Methods:**

We conducted a multicenter observational cohort study including 487 inpatients receiving standardized CGC at three hospitals in Germany. QoL was assessed using the WHOQOL-BREF global items (overall QoL and self-rated health). We examined (1) correlations between the global items and the four WHOQOL domains, (2) interrelations among variables using a regularized partial correlation network with bootstrap stability analysis, and (3) determinants of QoL using multiple linear regression with demographic, clinical, functional and psychosocial factors.

**Results:**

Regression analyses based solely on clinical-functional assessment explained only a small proportion of variance in QoL (adjusted R^2^ = 0.06). Adding sociodemographic and psychosocial factors substantially improved the model (adjusted R^2^ = 0.26), with depressive symptoms (β = –0.22), anxiety (β = –0.20), and self-efficacy (β = 0.17) emerging as the strongest predictors of QoL, while functional variables lost significance. Domain-specific regressions confirmed that psychosocial factors consistently predicted all WHOQOL subdomains. Network analysis revealed a cohesive QoL cluster with strong positive connections between QoL domains and negative connections from depressive symptoms, anxiety and loneliness. Self-efficacy emerged as a key positive node.

**Conclusion:**

Psychological distress (particularly depressive symptoms, anxiety, loneliness and reduced self-efficacy) was more strongly associated with QoL than traditional clinical-functional variables assessed in the CGA variables among older adults receiving CGC. Routine assessment of psychosocial burden and resources should be integrated into CGC to enable more individualized, patient-centered rehabilitation strategies.

**Supplementary Information:**

The online version contains supplementary material available at 10.1186/s12877-026-07728-9.

## Background

Quality of life (QoL) has emerged as a central outcome in geriatric medicine [1, 2]. It is increasingly considered not only a patient-centered endpoint but also an indicator of treatment effectiveness, functional prognosis, and long-term well-being [3]. To assess QoL in clinical and research settings, standardized patient-reported outcome measures are required that are both psychometrically sound and feasible in routine care. One of the most widely used instruments worldwide is the World Health Organization Quality of Life questionnaire (WHOQOL-BREF), a validated multidimensional measure capturing physical, psychological, social, and environmental aspects of QoL [4].

Older adults have specific medical, functional, and psychosocial needs, particularly in the context of acute or chronic illness [5]. Multimorbidity, functional decline, cognitive impairment, and social vulnerability often coexist and interact, requiring treatment approaches that go beyond disease-specific care and address the person as a whole [6]. These complex needs have led to the development of specialized geriatric care models that integrate medical, functional, and psychosocial perspectives [6]. In Germany, Comprehensive Geriatric Care (CGC) is provided by specialized hospitals. Combining medical treatment with functional rehabilitation and psychosocial support, it is delivered by multidisciplinary teams comprising physicians, nurses, physiotherapists, occupational therapists, speech and language therapists, psychologists and social workers. These teams work together to develop personalized rehabilitation plans aimed at improving especially physical functioning. CGC is defined within the Operations and Procedures Classification System (OPS) under code 8–550 [6]. Allowing reimbursement by statutory health insurance. The ability to perform activities of daily living is evaluated on a weekly basis as part of the CGC and in accordance with the specifications of the OPS 8–550 [7]. The Barthel Index is a suitable assessment tool for this purpose [8]. Consequently, it is the most commonly used instrument for assessing functional impairment on geriatric wards in Germany [9]. Furthermore, a comprehensive geriatric assessment (CGA) is required for all inpatients to determine their mobility, cognitive abilities, depression, and social functioning at the time of admission. This is due to an increase in the prevalence of these issues among older individuals. Research has consistently shown that CGC improves functional outcomes [10–13], and meta-analyses further demonstrate that both admission to acute geriatric units and the implementation of CGC result in superior clinical outcomes compared to standard hospital treatment [14, 15]. Recent findings indicate that, while depressive symptoms are significantly associated with numerous patient-centered outcomes, they do not impact functional improvement at the conclusion of CGC treatment [16]. In the context of this study, patient-centered outcomes are defined as self-reported measures reflecting patients’ subjective experiences, including QoL and psychosocial factors such as depressive symptoms, anxiety, and self-efficacy.

Despite its central relevance, QoL often remains a secondary or poorly operationalized endpoint in CGC, overshadowed by functional and care-process metrics. An important reason, besides the lack of necessity according to the OPS, is feasibility: even the WHOQOL-BREF, which is already the short form of the original 100-item WHOQOL, still comprises 26 items [17] and can be difficult to administer in routine clinical care, particularly in geriatric inpatient settings where time constraints, cognitive burden, and limited staff resources are common. Geriatric patients are often unable to complete these questionnaires independently [18]. Although the CGA routinely records a broad set of clinical and functional indicators, many of the factors most strongly influencing QoL, such as anxiety, loneliness, and self-efficacy [19–21], are not systematically assessed [9] Consequently, essential psychosocial determinants of QoL remain underrepresented in CGC, and treatment decisions rely predominantly on functional measures rather than on patient-reported well-being. This mismatch between what CGC routinely measures and what actually drives QoL represents a critical gap in current geriatric care.

Given this gap, the present study aimed to identify the clinical, functional, and psychosocial factors most strongly associated with QoL among older adults receiving CGC, with particular attention to variables routinely collected in the comprehensive geriatric assessment compared with additional psychosocial determinants. In addition, we aimed to evaluate whether the two WHOQOL-BREF global items (overall QoL and self-rated health) provide a valid and efficient summary measure of overall QoL by examining their correlations with the four WHOQOL-BREF domains and by comparing their explanatory value with domain-based QoL scales. Together, these aims seek to inform more patient-centered approaches to routine geriatric care by clarifying which domains contribute most to older adults’ perceived QoL.

## Methods

### Study design and population

Data on hospitalized geriatric patients was collected from the SelfManGer and JenaGer studies [22], which were conducted at three geriatric hospitals in Saxony-Anhalt (SelfManGer, trial registration DRKS00031016, 2023-04-05) and Thuringia (JenaGer, trial registration DRKS00032328, 2023-07-20). Although the SelfManGer and JenaGer studies had different overall aims, they were designed in concordance with overlapping instruments to ensure two comparable core datasets.

Data collection took place between February 2023 and August 2024. We included older inpatients aged 65 and older who received CGC within the German Operation and Procedure Classification System (OPS 8–550). The exclusion criteria were severe dementia or acute delirium, severe depression and full dependency on activities of daily living, as defined by a Barthel Index of 0 points [8]. Except for diagnosed dementia and acute delirium, we did not specify a cut-off point for cognitive tests as an exclusion. We included patients if study staff felt they were able to hold a meaningful conversation and comprehend the questionnaires [23]. All patients were screened based on their medical records. Then, trained study staff approached eligible patients, and they were informed about the study. If the patients gave their written informed consent, the study staff proceeded to go through the questionnaires together [23]. The study included both questionnaires and medical information obtained from the CGA performed as part of routine inpatient care. The full list of questionnaires is available in the data sheet [24].

The inclusion criteria for this specific analysis of the data required a complete measure of the WHOQOL-BREF without any missing data. Accordingly, 487 patients were included in further analyses.

#### Study size

The sample size was determined by the number of eligible patients recruited within the predefined study period across participating centers. The sample size is considered adequate for multivariable regression analyses based on established subject-to-variable ratios.

#### Bias considerations

Selection bias may have occurred due to the inclusion of patients able to provide informed consent and complete questionnaires, potentially leading to a healthier and cognitively less impaired sample. To mitigate this, inclusion criteria were kept broad and recruitment was conducted consecutively across multiple centers.

Information bias was minimized by using validated and standardized assessment instruments.

Missing data were handled using multiple imputation to reduce bias associated with incomplete observations. Sensitivity analyses using complete cases were conducted to assess robustness of results.

### Measures

All variables were defined a priori based on theoretical considerations and previous literature. We used the following variables, which were collected via paper-based, validated questionnaires.

#### Dependent variable:

The primary outcome was QoL as measured by the WHOQOL-BREF [4]. The WHOQOL-BREF was designed by the WHO and is a commonly used, validated measure of QoL, comprising four QoL domains: Physical (7 items), Psychological (6 items), Environment (8 items) and Social (3 items). Two additional item asks about overall QoL (“How would you rate your quality of life?”) and self-rated health (SRH) (“How satisfied are you with your health?”) combined to the global composite score, bringing the total number of items to 26. Question responses are recorded on a five-point Likert scale. To calculate the domain scores, the average response score for each domain was computed by dividing the total response score by the number of questions, and this result was multiplied by 4. The domain scores were then converted to a scale of 0 to 100 by subtracting 4 from each domain score and multiplying the difference by 6.25 [4]. In addition, the two global items (overall QoL and SRH) were combined and processed analogously to the domain scores to ensure comparability across outcome measures. A score of 0 represents the worst possible condition for the respective domain, while a score of 100 represents the best. The Cronbach’s alpha values for the domains of Physical, Psychological, Environment and Social in our data are 0.76, 0.70, 0.75, and 0.69, respectively, demonstrating good internal consistency.

It is important to distinguish between subjective satisfaction-based outcomes and objective or functional health outcomes. The WHOQOL-BREF primarily captures subjective perceptions of well-being and satisfaction with health, rather than objective functional status [4]. Accordingly, the questionnaire items are phrased to reflect personal evaluation and perceived quality rather than performance-based or clinical measures.

#### Covariates

Based on the literature and in accordance with the requirements of the OPS-8-550 [16], the following variables were selected as covariates from the clinical-functional assessment (CFA) within the framework of CGA (model 1):

- *age* (metric) in years,
- *sex* (female and male),
- cognition (Mini-Mental State Examination, *MMSE*, metric; higher values indicate better cognition) [25]: *Cronbach’s alpha* = 0.71,
- a performance-oriented mobility assessment (*Tinetti* test, metric; higher values indicate better mobility) [26]: *Cronbach’s alpha* = 0.90,
- activities of daily living (ADL) at baseline (*Barthel* Index, metric; higher values indicate better daily functioning) [8]: *Cronbach’s alpha* = 0.84, and
- Malnutrition (no risk and at risk/malnutrition): combination from short-form mini-nutritional assessment (*MNA)* [27] and Nutritional Risk Screening (*NRS*) [28].

In addition, we considered further psychosocial variables that are known to influence QoL in older people (model 2). These include:

- depressive symptoms (15-items Geriatric Depression Scale, *GDS*, metric; higher values indicate more depressive symptoms) [29]: *Cronbach’s alpha* = 0.70,
  The reduced quality of life associated with depressive symptoms results from a combination of psychological, somatic, social, and biological factors that directly and indirectly impair functioning and subjective well-being [30]. This assessment is routinely included in the CGA.

- loneliness [19] (short version [31] of the R-UCLA Loneliness Scale (revised *UCLA*) [32]): *Cronbach’s alpha* = 0.75; higher values indicate more loneliness
  Loneliness is an independent and significant risk factor for reduced QoL, particularly through the promotion of psychological stress and the deterioration of general well-being [19].

- self-efficacy [20] (*generalized self-efficacy scale* [33]): *Cronbach’s alpha* = 0.95; higher values indicate better self-efficacy
  People with strong self-efficacy demonstrate better coping skills, more initiative in dealing with symptoms and therapies, and greater resilience to stressors, which has a direct positive impact on QoL [34].

- anxiety [21] (5-item version of Beck Anxiety Inventory, *BAI* [35]): *Cronbach’s alpha* = 0.78; higher values indicate more anxiety.
  Anxiety is associated with QoL because it leads to considerable subjective stress, functional limitations, and psychosocial impairments [36].

- marital status [37] (married/with partner and single/widowed/divorced),
  On average, married people report less loneliness, more trust, and greater satisfaction in their social environment, which increases their subjective QoL [38].

- education [39] (low and middle/high). Low education attainment was defined as up to eight years of schooling. Medium and high educational attainment was defined as at least 10 years of schooling. The association between educational attainment and quality of life is multifactorial and robustly proven, with education considered a key social determinant of health and well-being [40]. The middle/high group is relatively homogeneous in terms of QoL, as there are usually no significant differences between middle and higher education among older people [41]. Therefore, these categories were combined.

### Statistical analyses

For statistical analyses, IBM SPSS statistics (Version 29) and R (Version 4.3.1) were used. The following packages were used: lme4 package (v1.1–26) [42], lmerTest (v3.2–1) [43], merTools (v0.6.4) [44], and sjPlot (v2.8.10) [45] for the linear mixed models and their stability analyses; bootnet (v1.8) [46] and qgraph (v1.9.8) [47] for network estimation, visualization, and stability analyses. Statistical significance for all tests was set at p < 0.05. Data were checked for normality using the Shapiro–Wilk test. Results were reported as median and interquartile ranges (IQR) for non-normally distributed data, and count and percentage for categorical variable. Although several variables deviated from normal distribution, means and standard deviations (SD) are additionally reported in accordance with common reporting standards in clinical and psychological research. The overall proportion of missing values was 0.2–7.4%. Little’s test for missing completely at random [48] was significant (χ^2^ = 451.47, df = 377, p = 0.005), indicating that the Missing Completely at Random assumption was not met. To assess the plausibility of the Missing at Random assumption, separate variance t tests were conducted to compare observed and missing groups. These analyses did not indicate systematic differences suggesting a violation of the Missing at Random assumption, supporting the use of multiple imputation methods as the primary analytical approach. Multiple imputations were conducted using Predictive Mean Matching under the Fully Conditional Specification framework. Five imputed datasets were generated using 10 iterations per dataset. Analyses based on complete cases using listwise deletion were conducted as sensitivity analyses, to assess the robustness of the findings with respect to the handling of missing data. Sensitivity analyses of the results from multiply imputed datasets are reported in the additional material (Additional Table 1/2). As quality control, we repeated the analyses with the different recruitment hospitals as an additional covariate. As the results indicated no significant influence of Hospital site on the relevant outcomes, for purposes of readability, we report only the hospital-overarching results in the main manuscript (Additional Table 3).

To examine whether the two WHOQOL-BREF global items (“overall quality of life” and “satisfaction with health”) adequately represent general QoL in our sample, we calculated Spearman’s rank correlations (ρ) between both global items and the four WHOQOL-BREF subdomains (physical, psychological, social, environmental) (Additional Table 4). Spearman’s correlation was chosen due to non-normal distribution of several QoL variables. Correlations were interpreted according to conventional thresholds (ρ ≥ 0.10 = weak, ≥ 0.30 = moderate, ≥ 0.50 = strong) [49].

To identify determinants of overall QoL, we conducted a series of multiple linear regression analyses using the scaled composite score of the two WHOQOL-BREF global items QoL and SRH as the dependent variable. All predictors were entered into the regression models simultaneously (enter method), and no variable selection procedures applied. In the first model, we included sociodemographic and clinical variables routinely assessed within the CFA (age, sex, Barthel Index, Tinetti score, cognitive status) (model 1). In a second model, we added psychosocial variables (depressive symptoms, anxiety, loneliness, self-efficacy, education, and marital status) to evaluate their incremental explanatory value beyond the CFA (model 2). To explore whether determinants differed across specific domains of QoL, we repeated the analyses using each of the four WHOQOL-BREF subscales (physical, psychological, social, environmental) as dependent variables. Assumptions for linear regression (linearity, homoscedasticity, multicollinearity, independence of residuals) were checked for all models. VIF-values were < 5 and tolerance > 0.1. Standardized coefficients (β), confidence interval (CI), and model fit indices were reported.

The network analysis was conducted as an exploratory, correlational visualization tool to complement the regression-based results and to illustrate the structural relationships among variables. The network was estimated using the EBICglasso method, which computes a sparse Gaussian graphical model based on regularized partial correlations. All variables were standardized prior to analysis. To assess the robustness of the network, we performed nonparametric bootstrapping with 1,000 resamples to evaluate the stability of edge weights and to obtain confidence intervals around the estimated edges. Additionally, the stability of node centrality (expected influence) was examined using case-dropping subset bootstrapping. The correlation stability coefficient (CS-coefficient) was calculated to quantify how strongly centrality estimates remained consistent when subsets of the data were removed. According to established guidelines, CS-coefficients above 0.50 indicate good stability [46].

## Results

### Baseline characteristics

A total of 1668 patients undergoing a CGC program (OPS 8–550) were screened for eligibility. Of these, 217 (13%) denied participation, 830 (50%) were excluded based on the exclusion criteria and 110 were unable to complete the assessment (7%), resulting in a final analytical sample of 511 patients. A further 24 patients (5%) were excluded due to incomplete QoL information, giving a final sample size of 487 [50]. The median age was 83 years (IQR 79–87), ranging from 67 to 96 years, and 66.1% of participants were female. Most had middle or high education (n = 467, 96.5%) and were either single, separated, or widowed (n = 313, 64.4%). Functional status was moderately impaired, with a median Barthel Index of 45 (IQR 35–60) and a median Tinetti score of 14 (IQR 9–19). Cognitive function was relatively preserved (MMSE median = 26, IQR 23–28). Psychosocial indicators showed low-to-moderate symptom levels (BAI median = 1; GDS median = 3; UCLA loneliness median = 3; SWE median = 30) (Table 1). Domain scores of the WHOQOL-BREF indicated moderate QoL, with median values ranging from 54 (Physical) to 75 (Environmental); the global QoL & SRH score had a median of 50 (IQR 38–62) (Additional Table 4).

**Table 1:** Sociodemographic characteristics

		*n*	%			Missings n (%)
Sex	female	322	66.1%			0
	male	165	33.9%			
Education	low	17	3.5%			3 (0.6%)
	middle/high	467	96.5%			
Marital status	single/separated/widowed	313	64.4%			1 (0.2%)
	Married/with partner	173	35.6%			
Malnutrition	no risk	175	40.1%			36 (7.4%)
	at risk/malnutrition	261	59.9%			
		Mean	SD	Median	IQR	
Age (in years)		83	6	83	79–87	0
Barthel		47	19	45	35–60	1 (0.2%)
Tinetti		14	7	14	9–19	27 (5.5%)
MMSE		25	3	26	23–28	4 (0.8%)
BAI		2	3	1	0–3	8 (1.6%)
UCLA		4	2	3	3–6	15 (3.1%)
SWE		30	7	30	26–35	25 (5.1%)
GDS		4	3	3	2–6	1 (0.2%)

*IQR* interquartile range, *SD* standard deviation, *BAI* Beck Anxiety Inventory, *GDS* Geriatric Depression Scale, *MMSE* Mini-mental state examination, *SWE* self-efficacy, *UCLA* Loneliness

Spearman correlations showed that the combined WHOQOL-BREF global score demonstrated broader and stronger associations with all QoL domains than either single item alone, correlating moderately to strongly with the physical (ρ = 0.51), psychological (ρ = 0.49), environmental (ρ = 0.31), and weakly with social domains (ρ = 0.18) (Additional Table 5). In contrast, item 1 (overall QoL) and item 2 (SRH) each showed narrower association patterns. Regression analyses confirmed this superiority of the combined score: the composite global score yielded the highest explained variance (Adj. R^2^ = 0.26) (Table 2), compared with item 1 (Adj. R^2^ = 0.19) (Additional Table 6) and item 2 (Adj. R^2^ = 0.18) (Additional Table 7). Therefore, in the main manuscript, results are reported for the composite QoL score consisting of Item 1 and 2. Full analyses of domain-specific models are reported in the Additional Table 8–11.

**Table 2:** Linear regression with variables from the CFA (model 1) and additional psychosocial variables (model 2)

Model		Unstandardized Coefficients		Beta	t	*p*	95% CI for B	
		B	SE				Lower	Upper
1	(Constant)	27.04	19.59		1.38	.17	-11.48	65.55
	age	.39	.19	.10	2.08	.04	.02	.77
	Sex—male	5.62	2.36	-.12	-2.38	.02	-10.26	-.98
	Malnutrition—yes	-.79	2.26	-.02	-.35	.73	-5.23	3.66
	MMSE	-.59	.34	-.09	-1.76	.08	-1.25	.07
	Barthel	.13	.07	.11	1.91	.06	-.01	.27
	Tinetti	.48	.18	.15	2.69	.01	.13	.83
	(1) Dependent Variable: WHO Subscale Quality of Life and Health – scaled *F*(6, 404) = 5.12, *p* < 0.001, Adjusted R2 = 0.06 Durbin-Watson 2.01							
2	(Constant)	14.79	24.66		.60	.55	-33.72	63.29
	age	.47	.18	.12	2.54	.01	.11	.83
	Sex—male	-5.98	2.33	-.12	-2.57	.01	-10.57	-1.40
	Malnutrition—yes	-.40	2.15	-.01	-.19	.85	-4.62	3.83
	MMSE	-.74	.32	-.11	-2.32	.02	-1.37	-.11
	Barthel	.11	.07	.09	1.70	.09	-.02	.24
	Tinetti	.55	.17	.17	3.26	.001	.22	.88
	GDS	-1.68	.37	-.22	-4.50	<.001	-2.41	-.94
	Education – middle/high	2.05	5.70	.02	.36	.72	-9.15	13.26
	Marital status—married	.63	2.34	.01	.27	.79	-3.97	5.24
	BAI	-1.62	.39	-.20	-4.14	<.001	-2.39	-.85
	UCLA	-.25	.55	-.02	-.47	.64	-1.33	.82
	SWE	.57	.17	.17	3.38	<.001	.24	.89
	(2) Dependent Variable: WHO Subscale Quality of Life and Health – scaled F(12, 364) = 11.78, p < 0.001, Adjusted R2 = 0.26 Durbin-Watson 1.89							

*SE* standard error, *p* significance, *CI* confidence interval, *BAI* Beck Anxiety Inventory, *GDS* Geriatric Depression Scale, *MMSE* Mini-mental state examination, *SWE* self-efficacy, *UCLA* Loneliness

### Determinants of QoL

In the first regression model including only the CFA, small effects were observed, with younger age, male sex, and better mobility (Tinetti score) significantly associated with higher QoL (Table 2: model 1). However, the overall explanatory power of these routine clinical and functional variables was low (adjusted R^2^ = 0.06), indicating that core CGA measures capture only a limited fraction of the variance in patients’ perceived QoL.

Adding psychosocial variables increased the explained variance to adjusted R^2^ = 0.26 (model 2), representing a marked shift in the determinant structure of QoL. The strongest predictors for lower QoL were reduced self-efficacy (B = 0.57, CI = 0.24–0.89, p < 0.001), anxiety (B = −1.62, CI = −2.39—−0.85, p < 0.001) and depressive symptoms (B = −1.68, CI = −2.41—−0.94, p < 0.001). Male gender, age, and mobility remained significant. Cognition became significant, but only in the analysis with list deletion and not in the imputed regressions (Additional Table 2). Accordingly, this effect was interpreted as not robust.

Regressions on the physical, psychological, social, and environmental subdomains (Additional Tables 8–11) further supported this pattern, showing that psychosocial factors—especially self-efficacy, depressive symptoms, anxiety, and loneliness—were the primary drivers of QoL across all domains. Functional and demographic variables showed only weak or non-significant contributions once psychosocial variables were taken into account. This increase in explained variance was statistically significant (ΔR^2^ = 0.19, *F-change*(6, 364) = 15.56, p < 0.001), formally confirming that the inclusion of psychosocial variables provides a significant incremental contribution beyond routine clinical and functional assessments.

**Fig. 1:**
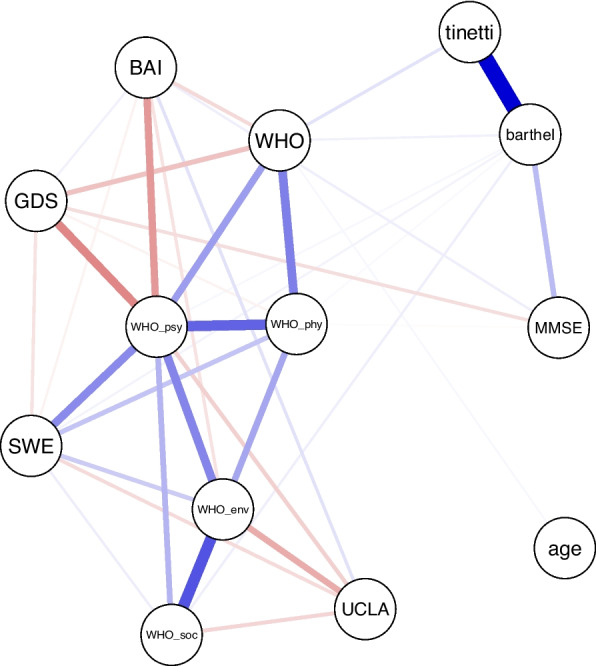
Network analysis. Blue edges indicate positive associations, red edges indicate negative associations between variables. Thicker edges represent stronger relationships. BAI: Beck Anxiety Inventory, GDS: Geriatric Depression Scale, MMSE: Mini-mental state examination, SWE: self-efficacy expectation, UCLA: Loneliness scale. WHO: WHOQOL-BREF Quality of life and self-rated health; WHO_env: WHOQOL-BREF environmental subdomain, WHO_phy: WHOQOL-BREF physical subdomain, WHO_psy: WHOQOL-BREF psychical subdomain, WHO_soc: WHOQOL-BREF social subdomain

For visualization purposes, we supplemented a psychometric network that exploratively depicts the interrelationships (Fig. 1). In the resulting network, the four WHOQOL-BREF domains formed a closely connected cluster, indicating substantial shared variance among physical, psychological, social, and environmental aspects of QoL after controlling for all other variables in the model. Depressive symptoms, anxiety, and loneliness showed negative partial associations with multiple QoL domains, whereas self-efficacy was positively associated with QoL domains. These associations reflect conditional relationships that persist when accounting for all remaining variables and should be interpreted as correlational rather than causal. Clinical and functional measures (e.g., ADL, mobility, cognition) showed comparatively fewer and weaker direct connections to QoL domains in the network structure. Overall, the network illustrates a pattern in which psychosocial variables are more densely connected to QoL domains than routine functional measures.

Stability analyses confirmed the robustness of the network. Bootstrapped confidence intervals around edge weights were narrow and sample–bootstrap curves closely overlapped (Additional Fig. 1), indicating high edge precision. The correlation stability coefficient for expected influence centrality was 0.75 (Additional Fig. 2) representing excellent stability.

## Discussion

This study examined factors associated with QoL in older adults undergoing CGC and evaluated whether routinely collected CGA variables sufficiently capture the factors most strongly related to perceived QoL. Two central findings emerged.

### The WHOQOL-BREF global items

The two WHOQOL-BREF global items (QoL and SRH) showed strong correlations with the physical and psychological domains, moderate correlation with the environmental domain and weak correlation with the social domain especially in comparison to the single items. Although the psychological domain demonstrated slightly stronger associations overall, the combination of the global items offer a practical, substantially less burdensome alternative, requiring only two items compared with six in the psychological domain [17]. Given that brevity and cognitive accessibility are critical in geriatric inpatient care, the global items appear to be valid and efficient summary indicators that are strongly associated with overall QoL. Beyond our empirical findings, the WHOQOL-BREF global items and their composite score are supported by extensive psychometric validation from the international WHO field trial [51], demonstrating good reliability, strong construct validity, and robust associations with all QoL domains across culturally diverse samples. This external evidence further supports the use of the combined global score as a valid summary indicator of overall QoL in older adults.

In contrast, disease-specific QoL measures offer greater sensitivity to disease-related symptoms and better responsiveness to clinical changes, whereas generic instruments allow for comparisons between disease groups and populations [52]. However, in a geriatric setting with a diverse patient population, QoL assessments generally seem more appropriate for capturing a broad range of domains applicable to various health conditions [53].

Additionally, previous research highlighting the utility of single-item QoL and health ratings in older populations due to their high convergent validity and reduced respondent burden [54, 55]. In terms of respondent burden, single- and two-item measures require significantly less time to complete and are less cognitively demanding, which is particularly advantageous in older populations who may experience fatigue or cognitive impairment. Studies have shown that response times can be reduced by up to 75% compared to multi-item instruments, while maintaining sufficient validity for group-level comparisons [56]. Emerging approaches such as computer adaptive testing (CAT) may further enhance the efficiency of patient-reported outcome assessment. It reduces the number of questions by an average of 45% without compromising measurement accuracy and significantly shortens the time required to complete the assessment, thereby greatly improving the efficiency of collecting patient-reported outcomes [57]. By tailoring item selection to individual response patterns, CAT has the potential to reduce response burden while maintaining or even improving measurement precision [58]. This may be particularly relevant in geriatric populations with limited cognitive or physical resources.

### Functional and psychological determinants of QoL

Our findings show that QoL during CGC is driven primarily by psychosocial factors rather than by traditional clinical or functional parameters. Depressive symptoms, anxiety, loneliness, and self-efficacy explained substantially more variance in QoL than mobility, ADL, cognition, or nutritional status. Functional capacity therefore appears necessary for independence and participation, but insufficient to explain how older adults evaluate their lives during illness and rehabilitation. This pattern can be explained within a stress–coping framework by Lazarus and Folkman [59]. Illness and functional impairment represent objective stressors, whereas QoL reflects the individual’s subjective appraisal of these stressors and perceived coping capacity. Psychological distress indicates maladaptive appraisal and emotional overload, while self-efficacy represents a central coping resource that buffers the negative impact of stressors. From this perspective, functional recovery provides the physical basis for autonomy, but psychological processes largely determine how this recovery is experienced and translated into perceived well-being.

Against this background, our results reveal a structural mismatch between what CGC routinely assesses and what determines QoL. The CGA and the OPS 8–550 framework primarily reflect a function-oriented rehabilitation paradigm, with a strong focus on mobility, ADL, and physical recovery [6, 16]. Although this focus is essential for restoring independence, it insufficiently captures psychosocial dimensions such as anxiety, loneliness, and coping resources, which our data show to be central drivers of QoL. By contrast, mobility, ADL, cognition and nutritional status accounted for only a small proportion of the variance once psychosocial variables were taken into account. As a result, routine CGC assessments systematically underrepresent domains that are most relevant for patient-reported well-being. These findings are consistent with previous research showing that loneliness, anxiety, and depressive symptoms are among the most powerful predictors of QoL in later life, often exceeding the influence of physical illness or functional impairment A recent systematic review and meta-analysis shows that depression (OR = 4.76), anxiety (OR = 5.10), and loneliness (OR = 3.30) are associated with significantly lower life satisfaction, and that these associations are stronger than those of physical limitations (OR = 2.64) [60]. The influence of loneliness is partly mediated by depressive symptoms [61]. Conversely, self-efficacy has repeatedly been shown to act as a protective resource that supports better coping, higher engagement in rehabilitation, and more positive self-evaluations of life circumstances [34]. Considering that psychosocial risk factors are modifiable through targeted interventions [62], integrating these assessments into CGC may offer clinically meaningful benefits. Routine identification of depressive symptoms, anxiety, loneliness, and low self-efficacy would allow clinicians to detect vulnerabilities that remain invisible in a primarily functional assessment framework. This, in turn, enables the timely initiation of evidence-based low-intensity psychological interventions, structured counseling, or targeted social support measures—approaches that have been shown to be feasible even in frail older populations and to yield substantial improvements in emotional well-being, treatment engagement, and overall QoL [63]. More broadly, incorporating psychosocial screening into CGC could strengthen interdisciplinary collaboration by giving psychologists, social workers, and nursing staff clearer entry points for intervention. It would also support the development of more individualized treatment pathways, in which psychosocial goals are explicitly defined alongside functional rehabilitation goals. Given that QoL is shaped more strongly by psychosocial factors than by traditional CGA metrics, not routinely assessing these domains may limit the ability to fully capture factors associated with patient-reported QoL. Brief screening of psychosocial burden may represent a feasible first step toward more patient-centered CGC without substantially increasing assessment burden.

This gap has major clinical relevance. More than 400,000 patients per year receive CGC in Germany [64], making it a central pillar of geriatric care. Although CGC effectively improves functional outcomes [6], the sustainability of its effects on patient-centered outcomes such as QoL is largely unknown. Our findings suggest that long-term benefit will not be achieved through functional rehabilitation alone but requires systematic attention to psychosocial determinants. If QoL is to be a core treatment goal, routine CGC should explicitly address anxiety, loneliness, depressive symptoms, and coping resources. In this context, the WHOQOL-BREF global items offer a practical solution. Their strong association with all QoL domains shows that overall QoL can be assessed reliably with only two items, making them highly suitable for routine geriatric inpatient care with limited time and cognitive capacity. Combined with brief screening of key psychosocial variables, they provide a feasible pathway toward more patient-centered, biopsychosocial CGC. Routinely collected QoL measures may also influence the doctor–patient dialogue in geriatric care. Systematic assessment of patient-reported outcomes can facilitate communication about subjective experiences that might otherwise remain underrecognized, particularly in the psychosocial domain [65]. This support more patient-centered communication, enhance shared decision-making, and help align clinical priorities with patients’ individual needs and preferences [66]. Effective communication represents the most proximal outcome in the care process. If QoL assessment doesn’t improve communication, effects on more distal outcomes like patient satisfaction are unlikely to occur [66]. In this regard, a well-being-focused and positive-oriented assessment can also be helpful.

Positive psychology interventions targeting constructs like flourishing, gratitude, optimism, and meaning demonstrate small to moderate effects on both wellbeing and symptom reduction in clinical populations. Importantly, these interventions not only improve wellbeing but can also reduce distress, suggesting dual benefits [67]. The Model for Sustainable Mental Health proposes systematic integration where both symptom reduction and wellbeing promotion are measured as distinct but complementary outcomes [68].

However, our results indicate that functional status alone appears to be insufficient to account for higher perceived QoL once psychological and psychosocial factors are considered. In this sense, functional capacity can be described as a necessary, but not sufficient, condition for higher QoL. From this perspective, QoL during CGC can be interpreted as the result of an interplay between functional prerequisites and psychological processes of stress appraisal and coping. While adequate functional capacity enables participation and independence, psychological distress may substantially undermine subjective well-being, whereas adaptive resources such as self-efficacy may buffer the impact of stressors and functional limitations on perceived QoL.

The discrepancy between the significant MMSE results in the main manuscript and the non-significant MMSE results in the multiple imputations can be explained by differences in how missing data was handled. Within this more representative sample, the effect of the MMSE becomes small and non-significant. This pattern is consistent with previous research suggesting that cognition is only weakly associated with QoL once affective and functional factors are considered [69].

The network analysis provided further support for the relevance of psychosocial determinants. Depressive symptoms, anxiety, and loneliness were negatively related to QoL domains, whereas higher self-efficacy was associated with higher QoL. These associations are consistent with the regression results. This structural pattern is consistent with a broader body of gerontological evidence emphasizing emotional well-being, perceived control, and social connectedness as core components associated with subjective well-being [70–72]. The network structure is consistent with this conceptualization, showing dense connections between indicators of psychological distress and multiple QoL domains, alongside positive associations between self-efficacy and QoL. This pattern supports the interpretation of QoL as embedded within a broader stress–coping context rather than being solely determined by functional status. The excellent stability of the network (CS = 0.75) shows that these associations are robust and unlikely to be sample-dependent. Although informative, the network should be interpreted as an exploratory and correlational visualization rather than a confirmatory analysis. Its purpose was to illustrate the structural pattern of associations complementing the regression results.

This study has several limitations that should be considered when interpreting its findings. Patients with severe depression were excluded from participation. This may have led to an underestimation of the association between depressive symptoms and QoL, as individuals with the highest burden of depression were not represented in the study sample. Furthermore, the inpatient setting itself may have influenced participants’ responses. Hospitalization is associated with multiple stressors, including the acute illness leading to admission, sleep disturbances, anxiety, disruption of daily routines, and separation from familiar environments and close social contacts [73]. These factors may have contributed to a more pessimistic self-assessment of QoL, potentially biasing the observed associations. These contextual influences should be considered when interpreting QoL as a situational rather than purely trait-like construct in this setting.

Although the regression analyses specify psychosocial variables as predictors of QoL and thus support a directional interpretation from depressive symptoms, anxiety, and self-efficacy to perceived QoL, the cross-sectional design does not allow us to exclude additional associations in the opposite direction. It therefore remains possible that reduced QoL also contributes to psychological burden, resulting in reciprocal or interactive effects that cannot be disentangled within the present study design. Longitudinal studies within CGC pathways are needed to clarify temporal relationships. Although the sample size was substantial, data were drawn from patients able to provide informed consent and participate in questionnaire-based assessment. This may have led to a healthier and more cognitively intact subsample of CGC inpatients (selection bias), limiting generalizability to individuals with advanced cognitive impairment, severe delirium or pronounced functional dependency. Finally, the network analysis, although robust and demonstrating high stability, reflects associations rather than causal processes, and node selection influences network topology. Replication in independent CGC cohorts and with alternative modelling approaches (e.g., mixed graphical models, longitudinal networks) would strengthen confidence in structural interpretations.

Despite these limitations, the study provides novel and clinically relevant insight into the psychosocial determinants of QoL in CGC and highlights assessment gaps with direct implications for geriatric care practice.

## Conclusions

Taken together, this study demonstrated that QoL during CGC is more strongly associated with psychosocial variables than with traditional functional or clinical factors. The strong performance of the WHOQOL global items further suggests that QoL can be captured efficiently without increasing assessment burden. Future CGC protocols may benefit from routine screening of psychosocial health, including anxiety, loneliness, and self-efficacy, to better detect needs that are not visible through functional assessments alone. Further research should explore how integrating such measures affects treatment outcomes, patient engagement, and long-term well-being.

## Supplementary Information

Additional file 1: Fig. 1. Bootstrap of Edge Weights. Bootstrap based on 1,000 nonparametric resamples. Black points represent the observed edge weights from the original sample, and grey confidence bands indicate the bootstrap variability.

Additional file 2: Fig. 2. Case-Dropping Bootstrap of expected influence. Bootstrap based on 1,000 nonparametric resamples. The plot depicts the correlation between original strength values and those obtained after progressively removing subsets of the sample. The correlation stability coefficient was 0.75.

Additional file 3: Table 1. Linear Regression with imputated data and CFA. Table 2. Linear Regression with imputated data and psychosocial variables. Table 3. Linear Regression with CFA (1) and psychosocial factors (2) with location. Table 4. Spearman’s Correlations. Table 5. Overview WHOQOL-BREF. Table 6. Linear Regression with WHOQOL-BREF Item 1 from the CFA (model 1) and additional psychosocial variables (model 2). Table 7. Linear Regression with WHOQOL-BREF Item 2 from the CFA (model 1) and additional psychosocial variables (model 2). Table 8. Linear Regression with WHO physical subscale and CFA (1) and psychosocial factors (2). Table 9. Linear Regression with WHO psychological subscale and CFA (1) and psychosocial factors (2). Table 10. Linear Regression with WHO social subscale and CFA (1) and psychosocial factors (2). Table 11. Linear Regression with WHO environmental subscale and standard assessment (1) and psychosocial factors (2).

## Data Availability

All study materials are available from Schönenberg A, Heimrich KG, Prell T. Data on Self-Management of Geriatric Syndromes. Open Science Framework. 2025 [24]. Of note, due to the potential sensitive nature of the data, the datasets are only available after request.

## Abbreviations

ADL: Activities of daily living
BAI: Beck Anxiety Inventory
CFA: Clinical-functional assessment
CGA: Comprehensive Geriatric Assessment
CGC: Comprehensive Geriatric Care
CI: Confidence interval
CS-coefficient: Correlation stability coefficient
GDS: Geriatric Depression Scale
IQR: Interquartile range
MMSE: Mini-Mental State Examination
OPS: Operations and Procedures Classification System
QoL: Quality of life
R-UCLA: Revised Loneliness Scale
SD: Standard deviation
SE: Standard error
SRH: Self-rated health
SWE: Self-efficacy
WHOQOL-BREF: World Health Organization Quality of Life questionnaire

## References

[1] Dinglas VD, Faraone LN, Needham DM. Understanding patient-important outcomes after critical illness: a synthesis of recent qualitative, empirical, and consensus-related studies. Curr Opin Crit Care. 2018;24(5):401–9. 30063492 10.1097/MCC.0000000000000533 PMC6133198

[2] Gajic O, Ahmad SR, Wilson ME, Kaufman DA. Outcomes of critical illness: what is meaningful? Curr Opin Crit Care. 2018;24(5):394–400. 30045089 10.1097/MCC.0000000000000530 PMC7008960

[3] Harinath G, Zalzala S, Nyquist A, Wouters M, Isman A, Moel M, et al. The role of quality of life data as an endpoint for collecting real-world evidence within geroscience clinical trials. Ageing Res Rev. 2024;97:102293. 38574864 10.1016/j.arr.2024.102293

[4] The WHOQOL Group. Development of the World Health Organization WHOQOL-BREF quality of life assessment. Psychol Med. 1998;28(3):551–8. 9626712 10.1017/s0033291798006667

[5] Prince MJ, Wu F, Guo Y, Gutierrez Robledo LM, O’Donnell M, Sullivan R, et al. The burden of disease in older people and implications for health policy and practice. Lancet. 2015;385(9967):549–62. 25468153 10.1016/S0140-6736(14)61347-7

[6] Heimrich KG, Lemhöfer C, Prell T. [Complex geriatric rehabilitation therapy]. Rehabilitation (Stuttg). 2025;64(3):176–84. 40494375 10.1055/a-2427-1414

[7] Geriatrie B. Auslegungshinweise des Bundesverbandes Geriatrie zum OPS 8–550 2024 [Available from: https://www.bv-geriatrie.de/images/INHALTE/Verbandsarbeit/DRG-Projektgruppe/Auslegungshinweise_2024/2024-Auslegungshinweise%20OPS%208-550_BV%20Geriatrie_final.pdf.

[8] Mahoney FI, Barthel DW. Functional evaluation: the Barthel index. Md State Med J. 1965;14:61–5. 14258950

[9] Kudelka J, Ollenschläger M, Dodel R, Eskofier BM, Hobert MA, Jahn K, et al. Which Comprehensive Geriatric Assessment (CGA) instruments are currently used in Germany: a survey. BMC Geriatr. 2024;24(1):347. 38627620 10.1186/s12877-024-04913-6 PMC11022468

[10] Niemöller U, Arnold A, Stein T, Juenemann M, Erkapic D, Rosenbauer J, et al. Comprehensive Geriatric Care in Older Adults: Walking Ability after an Acute Fracture. Med Sci (Basel). 2023;11(2):40. 10.3390/medsci11020040 PMC10301278 37367739

[11] Niemöller U, Arnold A, Stein T, Juenemann M, Farzat M, Erkapic D, et al. Comprehensive Geriatric Care in Older Hospitalized Patients with Depressive Symptoms. Geriatrics (Basel). 2023;8(2):37. 10.3390/geriatrics8020037 PMC10037575 36960992

[12] Kwetkat A, Lehmann T, Wittrich A. [Early geriatric rehabilitation: an opportunity for the oldest old]. Z Gerontol Geriatr. 2014;47(5):372–8. 24906436 10.1007/s00391-014-0660-7

[13] Werner C, Bauknecht L, Heldmann P, Hummel S, Günther-Lange M, Bauer JM, et al. Mobility outcomes and associated factors of acute geriatric care in hospitalized older patients: results from the PAGER study. Eur Geriatr Med. 2024;15(1):139–52. 37777992 10.1007/s41999-023-00869-9 PMC10876756

[14] O’Shaughnessy Í, Robinson K, O’Connor M, Conneely M, Ryan D, Steed F, et al. Effectiveness of acute geriatric unit care on functional decline, clinical and process outcomes among hospitalised older adults with acute medical complaints: a systematic review and meta-analysis. Age Ageing. 2022;51(4):afac081. 10.1093/ageing/afac081 PMC9053463 35486670

[15] Fox MT, Persaud M, Maimets I, O’Brien K, Brooks D, Tregunno D, et al. Effectiveness of acute geriatric unit care using acute care for elders components: a systematic review and meta-analysis. J Am Geriatr Soc. 2012;60(12):2237–45. 23176020 10.1111/jgs.12028 PMC3557720

[16] Heimrich KG, Schönenberg A, Mendorf S, Lehmann T, Prell T. Predictors of functional improvement during comprehensive geriatric care in Germany: a 10-year monocentric retrospective analysis. Sage Open Aging. 2025;11:30495334251346940. 40611850 10.1177/30495334251346941 PMC12220897

[17] Harper A, Power M. WHOQOL user manual. World Health Organisation; 1998.

[18] Siette J, Knaggs GT, Zurynski Y, Ratcliffe J, Dodds L, Westbrook J. Systematic review of 29 self-report instruments for assessing quality of life in older adults receiving aged care services. BMJ Open. 2021;11(11):e050892. 34794991 10.1136/bmjopen-2021-050892 PMC8603300

[19] Williams-Farrelly MM, Schroeder MW, Li C, Perkins AJ, Bakas T, Head KJ, et al. Loneliness in older primary care patients and its relationship to physical and mental health-related quality of life. J Am Geriatr Soc. 2024;72(3):811–21. 38240340 10.1111/jgs.18762 PMC10947914

[20] Lin CH, Liu CY, Huang CC, Rong JR. Frailty and quality of life among older adults in communities: the mediation effects of daily physical activity and healthy life self-efficacy. Geriatrics (Basel). 2022;7(6):125. 10.3390/geriatrics7060125 PMC9680389 36412614

[21] Sarma SI, Byrne GJ. Relationship between anxiety and quality of life in older mental health patients. Australas J Ageing. 2014;33(3):201–4. 25346973 10.1111/ajag.12102

[22] Prell T, Wientzek R, Schönenberg A. Self-management of geriatric syndromes - an observational study. BMC Geriatr. 2023;23(1):731. 37950176 10.1186/s12877-023-04442-8 PMC10638748

[23] Goodwin VA, Low MSA, Quinn TJ, Cockcroft EJ, Shepherd V, Evans PH, et al. Including older people in health and social care research: best practice recommendations based on the INCLUDE framework. Age Ageing. 2023;52(6):afad082. 10.1093/ageing/afad082 PMC10234283 37261448

[24] Schönenberg A, Heimrich KG, Prell T. Data on Self-Management of Geriatric Syndromes. Open Science Framework. 2025.

[25] Folstein MF, Folstein SE, McHugh PR. “Mini-mental state”. A practical method for grading the cognitive state of patients for the clinician. J Psychiatr Res. 1975;12(3):189–98. 1202204 10.1016/0022-3956(75)90026-6

[26] Tinetti ME. Performance-oriented assessment of mobility problems in elderly patients. J Am Geriatr Soc. 1986;34(2):119–26. 3944402 10.1111/j.1532-5415.1986.tb05480.x

[27] Rubenstein LZ, Harker JO, Salvà A, Guigoz Y, Vellas B. Screening for undernutrition in geriatric practice: developing the short-form mini-nutritional assessment (MNA-SF). J Gerontol A Biol Sci Med Sci. 2001;56(6):M366–72. 11382797 10.1093/gerona/56.6.m366

[28] Kondrup J, Rasmussen HH, Hamberg O, Stanga Z. Nutritional risk screening (NRS 2002): a new method based on an analysis of controlled clinical trials. Clin Nutr. 2003;22(3):321–36. 12765673 10.1016/s0261-5614(02)00214-5

[29] Yesavage J, Brink T, Rose T, Lum O, Huang V, Adey M, et al. Development and validation of a geriatric depression screening scale: a preliminary report. J Psychiatr Res. 1982;17(1):37–49. 7183759 10.1016/0022-3956(82)90033-4

[30] Tang AL, Thomas SJ. Relationships between depressive symptoms, other psychological symptoms, and quality of life. Psychiatry Res. 2020;289:113049. 32413710 10.1016/j.psychres.2020.113049

[31] Hughes ME, Waite LJ, Hawkley LC, Cacioppo JT. A short scale for measuring loneliness in large surveys: results from two population-based studies. Res Aging. 2004;26(6):655–72. 18504506 10.1177/0164027504268574 PMC2394670

[32] Russell D, Peplau LA, Ferguson ML. Developing a measure of loneliness. J Pers Assess. 1978;42(3):290–4. 660402 10.1207/s15327752jpa4203_11

[33] Schwarzer R, Jerusalem M. Skala zur Allgemeinen Selbstwirksamkeitserwartung [Verfahrensdokumentation, Autorenbeschreibung und Fragebogen]. Open Test Archive; 2003.

[34] Emamgholizadeh-Baboli E, Pashaei-Sabet F, Haghani H, Fotokian Z. Predicting the relationship of general self-efficacy and quality of life of the older adults with physical/mobility disabilities: a cross-sectional study in Northern Iran. BMC Geriatr. 2025;25(1):723. 41013331 10.1186/s12877-025-06348-z PMC12465195

[35] Beck AT, Epstein N, Brown G, Steer RA. An inventory for measuring clinical anxiety: psychometric properties. J Consult Clin Psychol. 1988;56(6):893–7. 3204199 10.1037//0022-006x.56.6.893

[36] Ribeiro O, Teixeira L, Araújo L, Rodríguez-Blázquez C, Calderón-Larrañaga A, Forjaz MJ. Anxiety, depression and quality of life in older adults: trajectories of influence across age. Int J Environ Res Public Health. 2020;17(23):9039. 10.3390/ijerph17239039 PMC7731150 33291547

[37] Gutiérrez-Vega M, Esparza-Del Villar OA, Carrillo-Saucedo IC, Montañez-Alvarado P. The possible protective effect of marital status in quality of life among elders in a U.S.-Mexico border city. Community Ment Health J. 2018;54(4):480–4. 28887605 10.1007/s10597-017-0166-z PMC5910460

[38] DeMaris A, Oates G. The trajectory of subjective well-being: a partial explanation of the marriage advantage. J Fam Issues. 2022;43(6):1650–68. 35755972 10.1177/0192513x211030033 PMC9230772

[39] Geigl C, Loss J, Leitzmann M, Janssen C. Social factors of health-related quality of life in older adults: a multivariable analysis. Qual Life Res. 2023;32(11):3257–68. 37458960 10.1007/s11136-023-03472-4 PMC10522508

[40] Newman DB, Gordon AM, Mendes WB. Income and education show distinct links to health and happiness in daily life. Nat Hum Behav. 2025;9(11):2299–312. 40781569 10.1038/s41562-025-02264-9 PMC13383670

[41] Głowacka M, Sienkiewicz Z, Dykowska G, Haor B. Dimensions of quality of life of older adults in relation to selected sociodemographic variables-a prospective cohort study. Front Public Health. 2024;12:1419008. 39363980 10.3389/fpubh.2024.1419008 PMC11447615

[42] Bates D, Mächler M, Bolker B, Walker S. Fitting linear mixed-effects models using lme4. J Stat Softw. 2015;67(1):1–48.

[43] Kuznetsova A, Brockhoff PB, Christensen RHB. lmerTest package: tests in linear mixed effects models. J Stat Softw. 2017;82(13):1–26.

[44] Knowles J, Frederick C. merTools: Tools for Analyzing Mixed Effect Regression Models https://github.com/jknowles/mertools2026.

[45] Lüdecke D. sjPlot: Data Visualization for Statistics in Social Science https://strengejacke.github.io/sjPlot/2021.

[46] Epskamp S, Borsboom D, Fried EI. Estimating psychological networks and their accuracy: A tutorial paper. Behav Res Methods. 2018;50(1):195–212. 28342071 10.3758/s13428-017-0862-1 PMC5809547

[47] Epskamp S, Cramer AOJ, Waldorp LJ, Schmittmann VD, Borsboom D. Qgraph: Network visualizations of relationships in psychometric data. J Stat Softw. 2012;48:1–18.

[48] Little R, Schenker N, G A, Cg C, Sobel M. Missing data. In: Handbook of statistical modeling for the social and behavioral sciences. Plenum Press; 1995. p. 39–75.

[49] Cohen J. Statistical power analysis for the behavioral sciences. L. Erlbaum Associates; 1988.

[50] Schönenberg A, Heimrich KG, Wientzek R, Berges N, Sternkopf A, Schindler A, et al. Self-Management of Geriatric Syndromes–longitudinal data on medical and psychosocial factors in older patients. Scientific Data. 2026;In revision. 10.1038/s41597-026-07405-x PMC13219512 42209528

[51] Skevington SM, Lotfy M, O’Connell KA. The World Health Organization’s WHOQOL-BREF quality of life assessment: psychometric properties and results of the international field trial. A report from the WHOQOL group. Qual Life Res. 2004;13(2):299–310. 15085902 10.1023/B:QURE.0000018486.91360.00

[52] Wiebe S, Guyatt G, Weaver B, Matijevic S, Sidwell C. Comparative responsiveness of generic and specific quality-of-life instruments. J Clin Epidemiol. 2003;56(1):52–60. 12589870 10.1016/s0895-4356(02)00537-1

[53] Testa MA, Simonson DC. Assessment of quality-of-life outcomes. N Engl J Med. 1996;334(13):835–40. 8596551 10.1056/NEJM199603283341306

[54] Fukui S, Ishikawa T, Iwahara Y, Fujikawa A, Fujita J, Takahashi K. Measuring well-being in older adults: Identifying an appropriate single-item questionnaire. Geriatr Gerontol Int. 2021;21(12):1131–7. 34697875 10.1111/ggi.14298

[55] Verster JC, Išerić E, Ulijn GA, Oskam SMP, Garssen J. Single-item assessment of quality of life: associations with well-being, mood, health correlates, and lifestyle. J Clin Med. 2024;13(17):5217. 10.3390/jcm13175217 PMC11396413 39274430

[56] Ware JE Jr. Improved items for estimating SF-36 profile and summary component scores: Construction and validation of an 8-Item QOL general (QGEN) survey. Med Care. 2025;63(4):300–10. 39823550 10.1097/MLR.0000000000002122 PMC11888827

[57] Lee D, Rao S, Campbell RE, Plummer OR, Tjoumakaris FP, Cohen SB, et al. The Application of Computerized Adaptive Testing to the International Knee Documentation Committee Subjective Knee Evaluation Form. Am J Sports Med. 2021;49(9):2426–31. 34161155 10.1177/03635465211021000

[58] Ridout K, Vanderlip E, Alter C, Carlo A, Kadriu B, Livesey C, et al. Resource document on implementation of measurement-based care. In: Association AP, editor. 2023. 10.1176/appi.ps.20240372 40471060

[59] Lazarus RS, Folkman S. Stress, appraisal, and coping. New York, NY: Springer Publishing Company; 1984.

[60] Sulandari S, Coats RO, Miller A, Hodkinson A, Johnson J. A systematic review and meta-analysis of the association between physical capability, social support, loneliness, depression, anxiety, and life satisfaction in older adults. Gerontologist. 2024;64(11):gnae128. 10.1093/geront/gnae128 PMC11512076 39233622

[61] Szeto JC, Fong DY, Kwok JY. Loneliness and its mediating relationship with depression, social support, and quality of life among long-term care facility residents. J Adv Nurs. 2025;0:1–13. 10.1111/jan.70343 41230728

[62] Wuthrich VM, Dickson SJ, Pehlivan M, Chen JT, Zagic D, Ghai I, et al. Efficacy of low intensity interventions for geriatric depression and anxiety - A systematic review and meta-analysis. J Affect Disord. 2024;344:592–9. 37858732 10.1016/j.jad.2023.10.093

[63] Emery-Tiburcio E, Zweig R, Brennan-Ing M, Sachs B, Shead V, Yenko I. APA guidlines for psychological practice with older adults. American Psychological Association; 2024. 10.1037/amp0001643 41396517

[64] (Destatis) SB. Fallpauschalenbezogene Krankenhausstatistik (DRG-Statistik) Operationen und Prozeduren der vollstationären Patientinnen und Patienten in Krankenhäusern (4-Steller) - 2023. 2024.

[65] Gibbons C, Porter I, Gonçalves-Bradley DC, Stoilov S, Ricci-Cabello I, Tsangaris E, et al. Routine provision of feedback from patient-reported outcome measurements to healthcare providers and patients in clinical practice. Cochrane Database Syst Rev. 2021;10(10):Cd011589. 34637526 10.1002/14651858.CD011589.pub2 PMC8509115

[66] Detmar SB, Muller MJ, Schornagel JH, Wever LDV, Aaronson NK. Health-related quality-of-life assessments and patient-physician communicationA randomized controlled trial. JAMA. 2002;288(23):3027–34. 12479768 10.1001/jama.288.23.3027

[67] Chakhssi F, Kraiss JT, Sommers-Spijkerman M, Bohlmeijer ET. The effect of positive psychology interventions on well-being and distress in clinical samples with psychiatric or somatic disorders: a systematic review and meta-analysis. BMC Psychiatry. 2018;18(1):211. 29945603 10.1186/s12888-018-1739-2 PMC6020379

[68] Bohlmeijer E, Westerhof G. The model for sustainable mental health: future directions for integrating Positive Psychology into mental health care. Front Psychol. 2021;12:747999. 34744925 10.3389/fpsyg.2021.747999 PMC8566941

[69] Black SA, Espino DV, Mahurin R, Lichtenstein MJ, Hazuda HP, Fabrizio D, et al. The influence of noncognitive factors on the Mini-Mental State Examination in older Mexican-Americans: findings from the Hispanic EPESE. Established Population for the Epidemiologic Study of the Elderly. J Clin Epidemiol. 1999;52(11):1095–102. 10.1016/s0895-4356(99)00100-6 10527004

[70] Lin FV, Simmons JM, Turnbull A, Zuo Y, Conwell Y, Wang KH. Cross-species framework for emotional well-being and brain aging: lessons from Behavioral Neuroscience. JAMA Psychiat. 2025;82(7):734–41. 10.1001/jamapsychiatry.2025.0581 PMC13508064 40332879

[71] Hong JH, Lachman ME, Charles ST, Chen Y, Wilson CL, Nakamura JS, et al. The positive influence of sense of control on physical, behavioral, and psychosocial health in older adults: An outcome-wide approach. Prev Med. 2021;149:106612. 33989673 10.1016/j.ypmed.2021.106612

[72] Chen Y, Wang D, Chen W, Zhao E, Li W, Zhu S, et al. Social capital, health status, and sociodemographic factors associated with subjective well-being among older adults: a comparative study of community dwellings and nursing homes. BMC Public Health. 2025;25(1):1259. 40181395 10.1186/s12889-025-22036-4 PMC11969848

[73] Rawal S, Kwan JL, Razak F, Detsky AS, Guo Y, Lapointe-Shaw L, et al. Association of the trauma of hospitalization with 30-day readmission or emergency department visit. JAMA Intern Med. 2019;179(1):38–45. 30508018 10.1001/jamainternmed.2018.5100 PMC6583419

