# Supplementary figures and images for: Psychological and functional factors associated with quality of life in comprehensive geriatric care: evidence from a multicenter observational cohort study
Source: BMC Geriatr. 2026 May 28;26:770. doi: 10.1186/s12877-026-07728-9 (PMC13220606; doi:10.1186/s12877-026-07728-9)

● Bootstrap mean    ● Sample

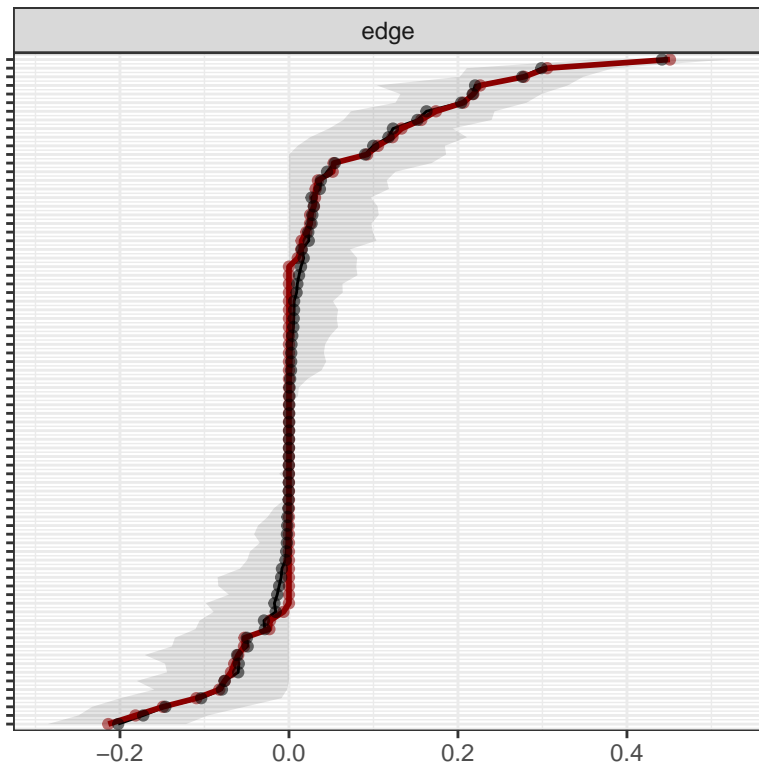

Supplement: Supplementary file 1 — Additional file 1: Fig. 1. Bootstrap of Edge Weights. Bootstrap based on 1,000 nonparametric resamples. Black points represent the observed edge weights from the original sample, and grey confidence bands indicate the bootstrap variability. [file 12877_2026_7728_MOESM1_ESM.pdf]

strength

Average correlation with original sample

Sampled cases

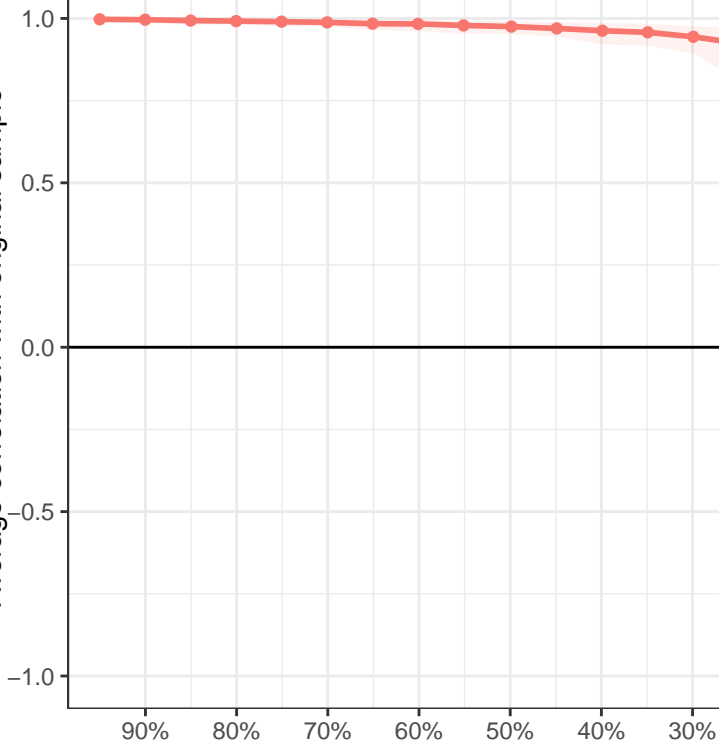

Supplement: Supplementary file 2 — Additional file 2: Fig. 2. Case-Dropping Bootstrap of expected influence. Bootstrap based on 1,000 nonparametric resamples. The plot depicts the correlation between original strength values and those obtained after progressively removing subsets of the sample. The correlation stability coefficient was 0.75. [file 12877_2026_7728_MOESM2_ESM.pdf]
