# Supplementary material for: Psychological and functional factors associated with quality of life in comprehensive geriatric care: evidence from a multicenter observational cohort study
Source: BMC Geriatr. 2026 May 28;26:770. doi: 10.1186/s12877-026-07728-9 (PMC13220606; doi:10.1186/s12877-026-07728-9)
Supplement: Supplementary file 3 — Additional file 3: Table 1. Linear Regression with imputated data and CFA. Table 2. Linear Regression with imputated data and psychosocial variables. Table 3. Linear Regression with CFA (1) and psychosocial factors (2) with location. Table 4. Spearman’s Correlations. Table 5. Overview WHOQOL-BREF. Table 6. Linear Regression with WHOQOL-BREF Item 1 from the CFA (model 1) and additional psychosocial variables (model 2). Table 7. Linear Regression with WHOQOL-BREF Item 2 from the CFA (model 1) and additional psychosocial variables (model 2). Table 8. Linear Regression with WHO physical subscale and CFA (1) and psychosocial factors (2). Table 9. Linear Regression with WHO psychological subscale and CFA (1) and psychosocial factors (2). Table 10. Linear Regression with WHO social subscale and CFA (1) and psychosocial factors (2). Table 11. Linear Regression with WHO environmental subscale and standard assessment (1) and psychosocial factors (2). [file 12877_2026_7728_MOESM3_ESM.docx]

**Additional Material**

**Table 1. Linear Regression with imputated data and CFA**

| Model | | Unstandardized Coefficients | | Beta | t | p | 95,0% CI for B | |
| --- | --- | --- | --- | --- | --- | --- | --- | --- |
|  |  | B | SE |  |  |  | Lower | Upper |
| 1 | (Constant) | 23.09 | 17.65 |  | 1.31 | .19 | -11.60 | 57.77 |
|  | age | .38 | .17 | .10 | 2.23 | **.03** | .04 | .71 |
|  | Sex - male | -4.82 | 2.14 | -.10 | -2.26 | **.02** | -9.02 | -.63 |
|  | Malnutrition - yes | .30 | 2.03 | .01 | .15 | .88 | -3.69 | 4.29 |
|  | MMSE | -.46 | .31 | -.07 | -1.45 | .14 | -1.06 | .14 |
|  | Barthel | .14 | .06 | .12 | 2.21 | **.03** | .02 | .26 |
|  | Tinetti | .46 | .17 | .14 | 2.77 | **.01** | .13 | .78 |
| 2 | (Constant) | 23.55 | 17.63 |  | 1.34 | .18 | -11.09 | 58.19 |
|  | age | .38 | .17 | .10 | 2.24 | **.03** | .05 | .71 |
|  | Sex - male | -4.76 | 2.13 | -.10 | -2.23 | **.03** | -8.94 | -.57 |
|  | Malnutrition - yes | .01 | 2.03 | .00 | .01 | .99 | -3.99 | 4.01 |
|  | MMSE | -.47 | .31 | -.07 | -1.53 | .13 | -1.07 | .13 |
|  | Barthel | .14 | .06 | .12 | 2.25 | **.03** | .02 | .26 |
|  | Tinetti | .44 | .17 | .14 | 2.67 | **.01** | .12 | .77 |
| 3 | (Constant) | 22.85 | 17.71 |  | 1.29 | .20 | -11.95 | 57.65 |
|  | age | .38 | .17 | .10 | 2.24 | **.03** | .05 | .71 |
|  | Sex - male | -4.82 | 2.13 | -.10 | -2.26 | **.02** | -9.01 | -.63 |
|  | Malnutrition - yes | .14 | 2.03 | .00 | .07 | .95 | -3.86 | 4.13 |
|  | MMSE | -.45 | .31 | -.07 | -1.45 | .15 | -1.05 | .16 |
|  | Barthel | .13 | .06 | .11 | 2.19 | **.03** | .01 | .26 |
|  | Tinetti | .46 | .17 | .14 | 2.78 | **.01** | .14 | .78 |
| 4 | (Constant) | 21.51 | 17.69 |  | 1.22 | .23 | -13.25 | 56.27 |
|  | age | .38 | .17 | .10 | 2.23 | **.03** | .05 | .71 |
|  | Sex - male | -4.76 | 2.13 | -.10 | -2.23 | **.03** | -8.94 | -.57 |
|  | Malnutrition - yes | .77 | 2.03 | .02 | .38 | .70 | -3.21 | 4.75 |
|  | MMSE | -.43 | .31 | -.07 | -1.41 | .16 | -1.04 | .17 |
|  | Barthel | .14 | .06 | .11 | 2.19 | **.03** | .01 | .26 |
|  | Tinetti | .45 | .17 | .14 | 2.75 | **.01** | .13 | .78 |
| 5 | (Constant) | 20.72 | 17.65 |  | 1.17 | .24 | -14.00 | 55.40 |
|  | age | .38 | .17 | .101 | 2.26 | **.03** | .05 | .71 |
|  | Sex - male | -4.72 | 2.13 | -.100 | -2.22 | **.03** | -8.90 | -.53 |
|  | Malnutrition - yes | 1.21 | 2.03 | .026 | .59 | .56 | -2.79 | 5.20 |
|  | MMSE | -.44 | .31 | -.066 | -1.45 | .15 | -1.05 | .16 |
|  | Barthel | .13 | .06 | .111 | 2.14 | **.03** | .01 | .25 |
|  | Tinetti | .47 | .17 | .146 | 2.87 | **.004** | .15 | .80 |
| Dependent Variable: WHO Subscale Quality of Life and Health – scaled  (1) *F*(6, 480) = 5.64, *p* < 0.001, Adjusted R2 = 0.05  Durbin-Watson 2.03  (2) *F*(6, 480) = 5.51, p < 0.001, Adjusted R2 = 0.05  Durbin-Watson 2.03  (3) *F*(6, 480) = 5.62, p < 0.001, Adjusted R2 = 0.05  Durbin-Watson 2.02  (4) *F*(6, 480) = 5.61, p < 0.001, Adjusted R2 = 0.05  Durbin-Watson 2.028  (5) *F*(6, 480) = 5.74, p < 0.001, Adjusted R2 = 0.06  Durbin-Watson 2.03  SE: standard error, p: significance, CI: confidence interval  GDS: Geriatric Depression Scale, MMSE: Mini-mental state examination | | | | | | | | |

**Table 2. Linear Regression with imputated data and psychosocial variables**

| Model | | Unstandardized Coefficients | | Beta | t | p | 95,0% CI for B | |
| --- | --- | --- | --- | --- | --- | --- | --- | --- |
|  |  | B | SE |  |  |  | Lower | Upper |
| 1 | (Constant) | 14.06 | 21.40 |  | .66 | .51 | -27.99 | 56.11 |
|  | age | .41 | .16 | .11 | 2.64 | **.01** | .11 | .72 |
|  | Sex - male | -4.98 | 2.06 | -.11 | -2.42 | **.02** | -9.02 | -.94 |
|  | Malnutrition - yes | .97 | 1.86 | .02 | .52 | .60 | -2.69 | 4.62 |
|  | MMSE | -.53 | .28 | -.08 | -1.85 | .07 | -1.09 | .03 |
|  | Barthel | .10 | .06 | .08 | 1.73 | .08 | -.01 | .21 |
|  | Tinetti | .44 | .15 | .14 | 2.93 | **.004** | .15 | .74 |
|  | GDS | -1.67 | .33 | -.23 | -5.11 | **<.001** | -2.32 | -1.03 |
|  | Education – middle/high | 2.85 | 5.01 | .02 | .57 | .57 | -6.99 | 12.68 |
|  | Marital status - married | .26 | 2.05 | .01 | .13 | .90 | -3.77 | 4.29 |
|  | BAI | -1.42 | .35 | -.18 | -4.10 | **<.001** | -2.09 | -.74 |
|  | UCLA | -.37 | .48 | -.03 | -.77 | .45 | -1.31 | .58 |
|  | SWE | .48 | .15 | .14 | 3.14 | **.002** | .18 | .78 |
| 2 | (Constant) | 15.16 | 21.46 |  | .71 | .48 | -27.02 | 57.33 |
|  | age | .42 | .16 | .11 | 2.65 | **.01** | .11 | .73 |
|  | Sex - male | -4.75 | 2.05 | -.10 | -2.31 | **.02** | -8.78 | -.71 |
|  | Malnutrition - yes | .91 | 1.87 | .02 | .49 | .63 | -2.76 | 4.58 |
|  | MMSE | -.53 | .29 | -.08 | -1.87 | .06 | -1.09 | .03 |
|  | Barthel | .11 | .06 | .09 | 1.85 | .06 | -.01 | .22 |
|  | Tinetti | .43 | .15 | .13 | 2.86 | **.004** | .14 | .73 |
|  | GDS | -1.69 | .33 | -.23 | -5.15 | **<.001** | -2.34 | -1.05 |
|  | Education – middle/high | 2.99 | 5.02 | .03 | .60 | .55 | -6.88 | 12.86 |
|  | Marital status - married | -.08 | 2.05 | -.00 | -.04 | .97 | -4.11 | 3.94 |
|  | BAI | -1.44 | .35 | -.18 | -4.16 | **<.001** | -2.12 | -.76 |
|  | UCLA | -.42 | .49 | -.04 | -.87 | .39 | -1.38 | .54 |
|  | SWE | .43 | .15 | .13 | 2.80 | **.01** | .13 | .73 |
| 3 | (Constant) | 11.03 | 21.38 |  | .52 | .61 | -30.97 | 53.04 |
|  | age | .43 | .16 | .12 | 2.73 | **.01** | .12 | .74 |
|  | Sex - male | -4.88 | 2.05 | -.10 | -2.38 | **.02** | -8.92 | -.85 |
|  | Malnutrition - yes | .68 | 1.86 | .02 | .37 | .72 | -2.98 | 4.34 |
|  | MMSE | -.51 | .28 | -.08 | -1.79 | .07 | -1.07 | .05 |
|  | Barthel | .10 | .06 | .08 | 1.74 | .08 | -.01 | .21 |
|  | Tinetti | .44 | .15 | .14 | 2.94 | **.003** | .15 | .74 |
|  | GDS | -1.69 | .33 | -.23 | -5.15 | **<.001** | -2.33 | -1.04 |
|  | Education – middle/high | 3.48 | 4.89 | .03 | .71 | .48 | -6.13 | 13.08 |
|  | Marital status - married | .25 | 2.05 | .01 | .12 | .90 | -3.78 | 4.28 |
|  | BAI | -1.38 | .35 | -.18 | -3.99 | **<.001** | -2.06 | -.70 |
|  | UCLA | -.34 | .49 | -.03 | -.71 | .48 | -1.30 | .61 |
|  | SWE | .48 | .15 | .14 | 3.18 | **.002** | .19 | .78 |
| 4 | (Constant) | 11.48 | 21.49 |  | .53 | .59 | -30.73 | 53.70 |
|  | age | .42 | .16 | .11 | 2.67 | **.01** | .11 | .73 |
|  | Sex - male | -4.84 | 2.05 | -.10 | -2.36 | **.02** | -8.87 | -.80 |
|  | Malnutrition - yes | 1.70 | 1.86 | .04 | .92 | .36 | -1.95 | 5.34 |
|  | MMSE | -.50 | .29 | -.07 | -1.75 | .08 | -1.06 | .06 |
|  | Barthel | .10 | .06 | .08 | 1.74 | .08 | -.01 | .21 |
|  | Tinetti | .45 | .15 | .14 | 2.97 | **.003** | .15 | .75 |
|  | GDS | -1.70 | .33 | -.23 | -5.19 | **<.001** | -2.35 | -1.06 |
|  | Education – middle/high | 3.15 | 5.01 | .03 | .63 | .53 | -6.69 | 12.99 |
|  | Marital status - married | .20 | 2.05 | .00 | .10 | .92 | -3.82 | 4.23 |
|  | BAI | -1.44 | .34 | -.18 | -4.19 | **<.001** | -2.12 | -.77 |
|  | UCLA | -.35 | .48 | -.03 | -.73 | .47 | -1.31 | .60 |
|  | SWE | .46 | .15 | .14 | 3.02 | **.003** | .16 | .76 |
| 5 | (Constant) | 10.75 | 21.41 |  | .50 | .62 | -31.31 | 52.82 |
|  | age | .42 | .16 | .11 | 2.67 | **.01** | .11 | .73 |
|  | Sex - male | -4.85 | 2.05 | -.10 | -2.36 | **.02** | -8.89 | -.82 |
|  | Malnutrition - yes | 2.21 | 1.86 | .05 | 1.19 | .24 | -1.45 | 5.88 |
|  | MMSE | -.51 | .28 | -.08 | -1.81 | .07 | -1.07 | .04 |
|  | Barthel | .09 | .06 | .08 | 1.66 | .10 | -.02 | .21 |
|  | Tinetti | .46 | .15 | .14 | 3.06 | **.002** | .17 | .76 |
|  | GDS | -1.72 | .33 | -.23 | -5.25 | **<.001** | -2.37 | -1.08 |
|  | Education – middle/high | 2.76 | 5.01 | .02 | .55 | .58 | -7.09 | 12.61 |
|  | Marital status - married | .29 | 2.06 | .01 | .14 | .89 | -3.75 | 4.33 |
|  | BAI | -1.35 | .35 | -.17 | -3.89 | **<.001** | -2.02 | -.67 |
|  | UCLA | -.31 | .48 | -.03 | -.64 | .52 | -1.26 | .64 |
|  | SWE | .48 | .15 | .14 | 3.19 | **.002** | .19 | .78 |
| Dependent Variable: WHO Subscale Quality of Life and Health – scaled  (1) *F*(12, 474) = 11.97, *p* < 0.001, Adjusted R2 = 0.21  Durbin-Watson 2.02  (2) *F*(12, 474) = 11.67, p < 0.001, Adjusted R2 = 0.21  Durbin-Watson 2.02  (3) *F*(12, 474) = 11.80, p < 0.001, Adjusted R2 = 0.21  Durbin-Watson 2.02  (4) *F*(12, 474) = 11.88, p < 0.001, Adjusted R2 = 0.21  Durbin-Watson 2.02  (5) *F*(12, 474) = 11.79, p < 0.001, Adjusted R2 = 0.21  Durbin-Watson 2.01  SE: standard error, p: significance, CI: confidence interval  BAI: Beck Anxiety Inventory, GDS: Geriatric Depression Scale, MMSE: Mini-mental state examination, SWE: self-efficacy expectation, UCLA: Loneliness scale. | | | | | | | | |

**Table 3. Linear Regression with CFA (1) and psychosocial factors (2) with location**

| Model | | Unstandardized Coefficients | | Beta | t | p | 95,0% CI for B | |
| --- | --- | --- | --- | --- | --- | --- | --- | --- |
|  |  | B | SE |  |  |  | Lower | Upper |
| 1 | (Constant) | 32.78 | 19.68 |  | 1.67 | .10 | -5.91 | 71.47 |
|  | age | .42 | .19 | .11 | 2.20 | **.03** | .04 | .79 |
|  | Sex - male | -5.37 | 2.35 | -.11 | -2.28 | **.02** | -9.99 | -.74 |
|  | Malnutrition - yes | -1.61 | 2.28 | -.04 | -.70 | .48 | -6.09 | 2.88 |
|  | MMSE | -.62 | .33 | -.09 | -1.86 | .06 | -1.28 | .04 |
|  | Barthel | .12 | .07 | .09 | 1.64 | **.10** | -.02 | .25 |
|  | Tinetti | .70 | .21 | .22 | 3.42 | **<.001** | .30 | 1.10 |
|  | Location | -3.96 | 1.83 | -.12 | -2.17 | **.03** | -7.55 | -.37 |
| 2 | (Constant) | 17.33 | 24.85 |  | .70 | .49 | -31.54 | 66.20 |
|  | age | .47 | .19 | .12 | 2.55 | **.01** | .11 | .83 |
|  | Sex - male | -5.79 | 2.34 | -.12 | -2.47 | **.01** | -10.40 | -1.18 |
|  | Malnutrition - yes | -.73 | 2.18 | -.02 | -.34 | .74 | -5.03 | 3.56 |
|  | MMSE | -.76 | .32 | -.11 | -2.35 | **.02** | -1.39 | -.13 |
|  | Barthel | .11 | .07 | .09 | 1.58 | .12 | -.03 | .24 |
|  | Tinetti | .62 | .19 | .19 | 3.29 | **.001** | .25 | 1.00 |
|  | GDS | -1.64 | .38 | -.21 | -4.35 | **<.001** | -2.38 | -.90 |
|  | Education – middle/high | 2.15 | 5.70 | .02 | .38 | .71 | -9.06 | 13.36 |
|  | Marital status - married | .36 | 2.36 | .01 | .15 | .88 | -4.29 | 5.01 |
|  | BAI | -1.58 | .39 | -.20 | -4.02 | **<.001** | -2.35 | -.81 |
|  | UCLA | -.32 | .55 | -.03 | -.58 | .57 | -1.40 | .77 |
|  | SWE | .57 | .17 | .17 | 3.42 | **<.001** | .24 | .90 |
|  | location | -1.53 | 1.79 | -.05 | -.86 | .39 | -5.05 | 1.99 |
| Dependent Variable: WHO Subscale Quality of Life and Health – scaled  *(1)F*(7, 403) = 5.10, *p* < 0.001, Adjusted R2 = 0.07  Durbin-Watson 2.02  (2) *F*(13, 363) = 10.92, *p* < 0.001, Adjusted R2 = 0.26  Durbin-Watson 1.89  SE: standard error, p: significance, CI: confidence interval  BAI: Beck Anxiety Inventory, GDS: Geriatric Depression Scale, MMSE: Mini-mental state examination, SWE: self-efficacy expectation, UCLA: Loneliness scale. | | | | | | | | |

**Table 4. Spearman’s Correlations**

|  | | WHO Item 1 (QoL) | WHO Item 2 (SRH) | WHO QoL & SRH | WHO Physical | WHO Psycho-logical | WHO Social | WHO Environ-mental |
| --- | --- | --- | --- | --- | --- | --- | --- | --- |
| WHO Item 1 (QoL) | ρ | 1.00 | **0.40*** | **0.79*** | **0.44*** | **0.47*** | **0.14*** | **0.32*** |
| WHO Item 2 (SRH) | ρ | **0.40*** | 1.00 | **0.87*** | **0.42*** | **0.37*** | **0.17*** | **0.21*** |
| WHO QoL & SRH | ρ | **0.79*** | **0.87*** | 1.00 | **0.51*** | **0.49*** | **0.18*** | **0.31*** |
| WHO Physical | ρ | **0.44*** | **0.42*** | **0.51*** | 1.00 | **0.62*** | **0.27*** | **0.46*** |
| WHO Psychological | ρ | **0.47*** | **0.37*** | **0.49*** | **0.62*** | 1.00 | **0.42*** | **0.58*** |
| WHO Social | ρ | **0.14*** | **0.17*** | **0.18*** | **0.27*** | **0.42*** | 1.00 | **0.52*** |
| WHO Environmental | ρ | **0.32*** | **0.21*** | **0.31*** | **0.46*** | **0.58*** | **0.52*** | 1.00 |
| * Correlation is significant at <0.001 level (2-tailed).  ρ: Spearman’s rank correlation coefficient  SRH: self-rated health, QoL: Quality of life, WHO: WHOQOL-BREF | | | | | | | | |

**Table 5. Overview WHOQOL-BREF**

|  | Mean | SD | Median | IQR |
| --- | --- | --- | --- | --- |
| WHO QoL & SRH | 50 | 22 | 50 | 38-62 |
| WHO Physical | 53 | 22 | 54 | 39-68 |
| WHO Psychological | 68 | 17 | 71 | 58-79 |
| WHO Social | 71 | 18 | 67 | 58-83 |
| WHO Environmental | 75 | 15 | 75 | 66-84 |
| SD: standard deviation, IQR: interquartile range  QoL: Quality of Life, SRH: self-rated health, WHO: WHOQOL-BREF | | | | |

**Table 6. Linear Regression with WHOQOL-BREF Item 1 from the CFA (model 1) and additional psychosocial variables (model 2)**

| Model | | Unstandardized Coefficients | | Beta | t | p | 95,0% CI for B | |
| --- | --- | --- | --- | --- | --- | --- | --- | --- |
|  |  | B | SE |  |  |  | Lower | Upper |
| 1 | (Constant) | -7.27 | 5.44 |  | -1.34 | .18 | -17.96 | 3.41 |
|  | age | .05 | .05 | .04 | .85 | .39 | -.06 | .15 |
|  | Sex - male | -1.10 | .66 | -.08 | -1.67 | .10 | -2.38 | .19 |
|  | Malnutrition - yes | -.49 | .63 | -.04 | -.78 | .44 | -1.72 | .75 |
|  | MMSE | -.08 | .09 | -.05 | -.90 | .37 | -.27 | .10 |
|  | Barthel | .04 | .02 | .13 | 2.22 | **.03** | .01 | .08 |
|  | Tinetti | .08 | .05 | .09 | 1.66 | .10 | -.02 | .18 |
| 2 | (Constant) | -15.44 | 6.94 |  | -2.23 | **.03** | -29.08 | -1.80 |
|  | age | .07 | .05 | .07 | 1.40 | .16 | -.03 | .18 |
|  | Sex - male | -1.35 | .66 | -.10 | -2.06 | **.04** | -2.64 | -.06 |
|  | Malnutrition - yes | -.38 | .60 | -.03 | -.62 | .53 | -1.57 | .81 |
|  | MMSE | -.10 | .09 | -.06 | -1.14 | .26 | -.28 | .08 |
|  | Barthel | .03 | .02 | .09 | 1.52 | .13 | -.01 | .07 |
|  | Tinetti | .12 | .05 | .14 | 2.63 | **.01** | .03 | .22 |
|  | GDS | -.30 | .11 | -.14 | -2.86 | **.01** | -.51 | -.09 |
|  | Education – middle/high | 2.15 | 1.60 | .06 | 1.34 | .18 | -1.01 | 5.30 |
|  | Marital status - married | .25 | .66 | .02 | .38 | .71 | -1.05 | 1.54 |
|  | BAI | -.45 | .11 | -.21 | -4.10 | **<.001** | -.66 | -.23 |
|  | UCLA | -.08 | .15 | -.03 | -.52 | .60 | -.38 | .22 |
|  | SWE | .17 | .05 | .18 | 3.50 | **<.001** | .07 | .26 |
| Dependent Variable: WHOQOL BREF Item 1 (QoL) - scaled  (1) *F*(6, 404) = 2.93, *p* = 0.01, Adjusted R2 = 0.03  Durbin-Watson 2.07  (2) F(12, 364) = 8.55, p < 0.001, Adjusted R2 = 0.19  Durbin-Watson 2.06  SE: standard error, p: significance, CI: confidence interval  BAI: Beck Anxiety Inventory, GDS: Geriatric Depression Scale, MMSE: Mini-mental state examination, SWE: self-efficacy expectation, UCLA: Loneliness scale. | | | | | | | | |

**Table 7. Linear Regression with WHOQOL-BREF Item 2 from the CFA (model 1) and additional psychosocial variables (model 2)**

| Model | | Unstandardized Coefficients | | Beta | t | p | 95,0% CI for B | |
| --- | --- | --- | --- | --- | --- | --- | --- | --- |
|  |  | B | SE |  |  |  | Lower | Upper |
| 1 | (Constant) | -16.71 | 6.17 |  | -2.71 | **.01** | -28.84 | -4.58 |
|  | age | .15 | .06 | .12 | 2.54 | **.01** | .03 | .27 |
|  | Sex - male | -1.71 | .74 | -.11 | -2.31 | **.02** | -3.18 | -.25 |
|  | Malnutrition - yes | .09 | .71 | .01 | .13 | .90 | -1.31 | 1.49 |
|  | MMSE | -.21 | .11 | -.10 | -2.01 | **.046** | -.42 | -.01 |
|  | Barthel | .02 | .02 | .06 | 1.08 | .28 | -.02 | .07 |
|  | Tinetti | .16 | .06 | .16 | 2.82 | **.01** | .05 | .27 |
| 2 | (Constant) | -14.67 | 8.13 |  | -1.80 | .07 | -30.66 | 1.33 |
|  | age | .16 | .06 | .13 | 2.66 | **.01** | .04 | .28 |
|  | Sex - male | -1.64 | .77 | -.11 | -2.14 | **.03** | -3.15 | -.13 |
|  | Malnutrition - yes | .18 | .71 | .01 | .25 | .80 | -1.21 | 1.57 |
|  | MMSE | -.27 | .11 | -.13 | -2.54 | **.01** | -.48 | -.06 |
|  | Barthel | .03 | .02 | .07 | 1.28 | .20 | -.02 | .07 |
|  | Tinetti | .15 | .06 | .15 | 2.71 | **.01** | .04 | .26 |
|  | GDS | -.54 | .12 | -.22 | -4.38 | **<.001** | -.78 | -.30 |
|  | Education – middle/high | -1.12 | 1.88 | -.03 | -.60 | .55 | -4.82 | 2.58 |
|  | Marital status - married | .07 | .77 | .01 | .09 | .93 | -1.45 | 1.59 |
|  | BAI | -.36 | .13 | -.15 | -2.80 | **.01** | -.61 | -.11 |
|  | UCLA | -.05 | .18 | -.01 | -.26 | .79 | -.40 | .31 |
|  | SWE | .12 | .06 | .11 | 2.15 | **.03** | .01 | .23 |
| Dependent Variable: WHOQOL BREF Item 2 (SRH) - scaled  (1) *F*(6, 404) = 4.87, *p* < 0.001, Adjusted R2 = 0.05  Durbin-Watson 2.02  (2) F(12, 364) = 7.90, p < 0.001, Adjusted R2 = 0.18  Durbin-Watson 2.09  SE: standard error, p: significance, CI: confidence interval  BAI: Beck Anxiety Inventory, GDS: Geriatric Depression Scale, MMSE: Mini-mental state examination, SWE: self-efficacy expectation, UCLA: Loneliness scale. | | | | | | | | |

**Table 8. Linear Regression with WHO physical subscale and CFA (1) and psychosocial factors (2)**

| Model | | Unstandardized Coefficients | | Beta | t | p | 95,0% CI for B | |
| --- | --- | --- | --- | --- | --- | --- | --- | --- |
|  |  | B | SE |  |  |  | Lower | Upper |
| 1 | (Constant) | 62.78 | 19.34 |  | 3.25 | **.001** | 24.75 | 100.81 |
|  | age | .09 | .19 | .03 | .50 | .62 | -.27 | .46 |
|  | Sex - male | -1.00 | 2.32 | -.02 | -.43 | .67 | -5.57 | 3.56 |
|  | Malnutrition - yes | -2.27 | 2.22 | -.05 | -1.02 | .31 | -6.63 | 2.10 |
|  | MMSE | -.99 | .33 | -.16 | -3.03 | **.003** | -1.64 | -.35 |
|  | Barthel | .20 | .07 | .17 | 2.96 | **.003** | .07 | .34 |
|  | Tinetti | .21 | .18 | .07 | 1.17 | .24 | -.14 | .56 |
| 2 | (Constant) | 25.80 | 23.99 |  | 1.08 | .28 | -21.39 | 72.99 |
|  | age | .16 | .18 | .04 | .87 | .38 | -.20 | .51 |
|  | Sex - male | -1.70 | 2.28 | -.04 | -.75 | .46 | -6.18 | 2.77 |
|  | Malnutrition - yes | -1.47 | 2.09 | -.03 | -.70 | .48 | -5.58 | 2.64 |
|  | MMSE | -.88 | .31 | -.13 | -2.82 | **.01** | -1.49 | -.27 |
|  | Barthel | .12 | .06 | .10 | 1.92 | .06 | -.01 | .25 |
|  | Tinetti | .26 | .17 | .08 | 1.58 | .11 | -.06 | .59 |
|  | GDS | -1.70 | .36 | -.23 | -4.71 | **<.001** | -2.41 | -.99 |
|  | Education – middle/high | 6.62 | 5.49 | .06 | 1.21 | .23 | -4.17 | 17.42 |
|  | Marital status - married | 1.21 | 2.28 | .03 | .53 | .60 | -3.27 | 5.69 |
|  | BAI | -1.16 | .38 | -.15 | -3.02 | **.003** | -1.91 | -.40 |
|  | UCLA | -.22 | .53 | -.02 | -.42 | .67 | -1.26 | .82 |
|  | SWE | .93 | .16 | .29 | 5.69 | **<.001** | .61 | 1.25 |
| Dependent Variable: WHO physical subscale – scaled  *(1)F*(6, 388) = 3.75, *p* = 0.001, Adjusted R2 = 0.04  Durbin-Watson 1.91  (2) *F*(12, 354) = 12.38, *p* < 0.001, Adjusted R2 = 0.30  Durbin-Watson 2.01  SE: standard error, p: significance, CI: confidence interval  BAI: Beck Anxiety Inventory, GDS: Geriatric Depression Scale, MMSE: Mini-mental state examination, SWE: self-efficacy expectation, UCLA: Loneliness scale. | | | | | | | | |

**Table 9. Linear Regression with WHO psychological subscale and CFA (1) and psychosocial factors (2)**

| Model | | Unstandardized Coefficients | | Beta | t | p | 95,0% CI for B | |
| --- | --- | --- | --- | --- | --- | --- | --- | --- |
|  |  | B | SE |  |  |  | Lower | Upper |
| 1 | (Constant) | 39.28 | 14.82 |  | 2.65 | **.01** | 10.15 | 68.41 |
|  | age | .29 | .14 | .10 | 2.01 | **.046** | .01 | .56 |
|  | Sex - male | -.30 | 1.78 | -.01 | -.17 | .87 | -3.79 | 3.20 |
|  | Malnutrition - yes | -2.10 | 1.69 | -.06 | -1.24 | .21 | -5.43 | 1.23 |
|  | MMSE | .02 | .26 | .01 | .08 | .94 | -.49 | .53 |
|  | Barthel | .23 | .05 | .25 | 4.38 | **<.001** | .13 | .34 |
|  | Tinetti | -.21 | .14 | -.09 | -1.53 | .13 | -.48 | .06 |
| 2 | (Constant) | 27.41 | 15.32 |  | 1.79 | .07 | -2.72 | 57.55 |
|  | age | .29 | .12 | .10 | 2.53 | **.01** | .07 | .52 |
|  | Sex - male | -.02 | 1.46 | -.01 | -.01 | .99 | -2.88 | 2.85 |
|  | Malnutrition - yes | -1.57 | 1.33 | -.05 | -1.18 | .24 | -4.19 | 1.05 |
|  | MMSE | -.07 | .20 | -.01 | -.35 | .73 | -.48 | .33 |
|  | Barthel | .16 | .04 | .17 | 3.76 | **<.001** | .08 | .24 |
|  | Tinetti | -.21 | .11 | -.09 | -1.93 | .05 | -.41 | .01 |
|  | GDS | -1.38 | .23 | -.25 | -5.98 | **<.001** | -1.84 | -.93 |
|  | Education – middle/high | 3.78 | 3.51 | .04 | 1.08 | .28 | -3.12 | 10.68 |
|  | Marital status - married | -.39 | 1.49 | -.01 | -.27 | .79 | -3.26 | 2.48 |
|  | BAI | -1.44 | .25 | -.25 | -5.89 | **<.001** | -1.92 | -.96 |
|  | UCLA | -1.25 | .34 | -.15 | -3.69 | **<.001** | -1.91 | -.58 |
|  | SWE | .78 | .10 | .32 | 7.53 | **<.001** | .58 | .98 |
| Dependent Variable: WHO psychological subscale – scaled  *(1)F*(6, 390) = 4.46, *p* < 0.001, Adjusted R2 = 0.05  Durbin-Watson 1.92  (2) *F*(12, 355) = 27.95, *p* < 0.001, Adjusted R2 = 0.47  Durbin-Watson 1.84  SE: standard error, p: significance, CI: confidence interval  BAI: Beck Anxiety Inventory, GDS: Geriatric Depression Scale, MMSE: Mini-mental state examination, SWE: self-efficacy expectation, UCLA: Loneliness scale. | | | | | | | | |

**Table 10. Linear Regression with WHO social subscale and CFA (1) and psychosocial factors (2)**

| Model | | Unstandardized Coefficients | | Beta | t | p | 95,0% CI for B | |
| --- | --- | --- | --- | --- | --- | --- | --- | --- |
|  |  | B | SE |  |  |  | Lower | Upper |
| 1 | (Constant) | 47.53 | 16.79 |  | 2.831 | **.01** | 14.51 | 80.54 |
|  | age | .01 | .16 | .01 | .04 | .97 | -.31 | .33 |
|  | Sex - male | -1.45 | 1.99 | -.04 | -.73 | .47 | -5.37 | 2.48 |
|  | Malnutrition - yes | .32 | 1.91 | .01 | .17 | .87 | -3.44 | 4.08 |
|  | MMSE | .63 | .29 | .11 | 2.14 | **.03** | .05 | 1.20 |
|  | Barthel | .16 | .06 | .16 | 2.70 | **.01** | .04 | .28 |
|  | Tinetti | -.02 | .16 | -.01 | -.14 | .89 | -.33 | .29 |
| 2 | (Constant) | 23.48 | 20.90 |  | 1.12 | .26 | -17.64 | 64.59 |
|  | age | .04 | .16 | .01 | .26 | .79 | -.27 | .35 |
|  | Sex - male | -1.27 | 1.97 | -.03 | -.64 | .52 | -5.15 | 2.62 |
|  | Malnutrition - yes | .75 | 1.81 | .02 | .42 | .68 | -2.81 | 4.32 |
|  | MMSE | .27 | .28 | .05 | .94 | .35 | -.29 | .83 |
|  | Barthel | .15 | .06 | .15 | 2.55 | .**01** | .03 | .26 |
|  | Tinetti | -.11 | .15 | -.04 | -.75 | .45 | -.40 | .18 |
|  | GDS | -.60 | .32 | -.10 | -1.90 | .06 | -1.23 | .02 |
|  | Education – middle/high | 13.33 | 4.71 | .14 | 2.83 | **.01** | 4.07 | 22.59 |
|  | Marital status - married | 1.86 | 1.97 | .05 | .95 | .35 | -2.02 | 5.74 |
|  | BAI | -.31 | .33 | -.05 | -.93 | .35 | -.96 | .34 |
|  | UCLA | -1.81 | .46 | -.21 | -3.94 | **<.001** | -2.72 | -.91 |
|  | SWE | .53 | .14 | .20 | 3.74 | **<.001** | .25 | .81 |
| Dependent Variable: WHO social subscale – scaled  *(1)F*(6, 377) = 3.02, *p* = 0.007, Adjusted R2 = 0.03  Durbin-Watson 1.95  (2) *F*(12, 344) = 7.88, *p* < 0.001, Adjusted R2 = 0.19  Durbin-Watson 2.09  SE: standard error, p: significance, CI: confidence interval  BAI: Beck Anxiety Inventory, GDS: Geriatric Depression Scale, MMSE: Mini-mental state examination, SWE: self-efficacy expectation, UCLA: Loneliness scale. | | | | | | | | |

**Table 11. Linear Regression with WHO environmental subscale and standard assessment (1) and psychosocial factors (2)**

| Model | | Unstandardized Coefficients | | Beta | t | p | 95,0% CI for B | |
| --- | --- | --- | --- | --- | --- | --- | --- | --- |
|  |  | B | SE |  |  |  | Lower | Upper |
| 1 | (Constant) | 73.50 | 13.37 |  | 5.50 | **<.001** | 47.23 | 99.78 |
|  | age | -.05 | .13 | -.02 | -.35 | .73 | -.30 | .21 |
|  | Sex - male | -.24 | 1.61 | -.01 | -.15 | .88 | -3.40 | 2.92 |
|  | Malnutrition - yes | -.97 | 1.54 | -.03 | -.63 | .53 | -3.99 | 2.05 |
|  | MMSE | .05 | .23 | .01 | .20 | .84 | -.40 | .49 |
|  | Barthel | .10 | .05 | .12 | 2.02 | **.04** | .01 | .19 |
|  | Tinetti | .03 | .12 | .02 | .26 | .79 | -.21 | .27 |
| 2 | (Constant) | 69.92 | 15.90 |  | 4.40 | **<.001** | 38.64 | 101.19 |
|  | age | -.08 | .12 | -.03 | -.66 | .51 | -.31 | .16 |
|  | Sex - male | -.62 | 1.51 | -.02 | -.41 | .68 | -3.59 | 2.36 |
|  | Malnutrition - yes | -.41 | 1.39 | -.01 | -.29 | .77 | -3.13 | 2.32 |
|  | MMSE | .01 | .21 | .01 | .02 | .99 | -.40 | .41 |
|  | Barthel | .05 | .04 | .07 | 1.21 | .23 | -.03 | .14 |
|  | Tinetti | .01 | .11 | .01 | .09 | .93 | -.21 | .23 |
|  | GDS | -.38 | .24 | -.08 | -1.56 | .12 | -.85 | .10 |
|  | Education – middle/high | 2.64 | 3.64 | .03 | .73 | .47 | -4.52 | 9.80 |
|  | Marital status - married | .08 | 1.51 | .01 | .05 | .96 | -2.90 | 3.05 |
|  | BAI | -1.01 | .26 | -.20 | -3.97 | **<.001** | -1.51 | -.51 |
|  | UCLA | -1.85 | .35 | -.26 | -5.23 | **<.001** | -2.55 | -1.16 |
|  | SWE | .54 | .11 | .25 | 5.00 | **<.001** | .33 | .75 |
| Dependent Variable: WHO environmental subscale – scaled  *(1)F*(6, 388) = 1.17, *p* = 0.32, Adjusted R2 = 0.003  Durbin-Watson 1.90  (2) *F*(12, 355) = 12.76, *p* < 0.001, Adjusted R2 = 0.28  Durbin-Watson 1.97  SE: standard error, p: significance, CI: confidence interval  BAI: Beck Anxiety Inventory, GDS: Geriatric Depression Scale, MMSE: Mini-mental state examination, SWE: self-efficacy expectation, UCLA: Loneliness scale. | | | | | | | | |
